# Hepatitis B infection and intrahepatic cholestasis of pregnancy: A systematic review and meta-analysis

**DOI:** 10.1097/MD.0000000000021416

**Published:** 2020-07-31

**Authors:** Ruoan Jiang, Ting Wang, Yingsha Yao, Feifei Zhou, Xiufeng Huang

**Affiliations:** Department of Obstetrics and Gynecology, Women's Hospital, Zhejiang University of Medicine, Hangzhou, Zhejiang Province, China.

**Keywords:** hepatitis B, intrahepatic cholestasis of pregnancy, meta-analysis, systematic review

## Abstract

**Background:**

Viral hepatitis type B is caused by hepatitis B virus (HBV) infection. Several studies have linked HBV infection to a higher risk of developing intrahepatic cholestasis of pregnancy (ICP), although some give contradictory results. To investigate the association and estimated risk of ICP in patients with HBV infection, we conducted this meta-analysis to summarize all available evidence.

**Methods:**

This study consists of 2 meta-analyses. A literature search was performed using MEDLINE and EMBASE from inception to July 2019. The first study included studies that reported associations between HBV infection and the risk of ICP. The second analysis included studies comparing the risk of HBV infection in ICP patients with those without ICP. Odds ratios (OR) and 95% confidence intervals (CI) were calculated using a random-effect, inverse variance method.

**Results:**

Four studies were included in both analyses. The OR of ICP in HBV-infected pregnant women compared with non-HBV pregnant women was 1.68 (95% CI 1.43–1.97; I^2^ = 0%). The OR of HBV infection among ICP patients compared with non-ICP patients was 1.70 (95% CI 1.44–2.01; I^2^ = 0%).

**Conclusions:**

Our meta-analysis demonstrates not only a higher risk of ICP among HBV-infected pregnant women but also an increased risk of HBV infection among ICP patients. These findings suggest that HBV is a high-risk factor for ICP and screening for hepatitis B in women with ICP symptoms may be beneficial.

## 1 Introduction

Hepatitis B infection is caused by hepatitis B virus (HBV). Chronic hepatitis B infection encompasses a spectrum of disease, and is defined as persistent HBV infection (the presence of detectable hepatitis B surface antigen [HBsAg] in the blood or serum for longer than 6 months), with or without associated active viral replication and evidence of hepatocellular injury and inflammation.^[1]^ HBV belongs to the family Hepadnaviridae and has a high infection rate, with approximately 600,000 patients dying of the disease worldwide annually. HBV infection has become an uncontrollable public health problem in developing countries. The prevalence of HBV infection among women of childbearing age may be as high as 2% to 8% in China,^[2,3]^ whereas it is only 0.4% in the United States.^[4]^ Worldwide, an estimated 2 billion people have evidence of past or present HBV infection, and 240 million are chronic carriers of HBsAg.^[5,6]^ China has a high incidence of hepatitis B infection. The positive rate of serum HBsAg is 10% to 15%, and the accumulated HBV infection rate is 60% to 70% in China.^[7–9]^

Intrahepatic cholestasis of pregnancy (ICP) has also been called recurrent jaundice of pregnancy, cholestatic hepatosis, and icterus gravidarum and is characterised by pruritus, icterus, or both.^[10]^ It is the most common liver disease during pregnancy, characterized by otherwise unexplained pruritus in the last half of pregnancy and elevated bile acids or transaminases.^[11,12]^ ICP is associated with an increased risk of adverse perinatal outcomes for the foetus, an increased risk of spontaneous preterm labour, foetal hypoxia, stillbirth, meconium-stained amniotic fluid, extended neonatal unit admission, perinatal death, and the late development of hepatobiliary disease in the mother.^[13]^ The incidence of ICP varies and is dependent on geographic location and ethnicity. For example, the incidence is 4% in Chile;^[14]^ 0.7% in the United Kingdom, where it is higher in women of Indian or Pakistani origin,^[15]^ and 1.2% in China based on more than 100,000 hospital births.^[16,17]^

HBV infection triggers an autoimmune response, and ultimately leads to hepatic cell damage. Moreover, specific hormone level changes during pregnancy can place an extra burden on the liver and aggravate liver disease. Although several studies have demonstrated that HBV infection is not a cause of excessive maternal morbidity and mortality and has no effect on fetal growth restriction or preeclampsia rates,^[18–20]^ other studies have found that HBV infection is associated with a higher incidence of adverse pregnancy outcomes, such as gestational diabetes mellitus,^[21]^ hypertensive disorders of pregnancy,^[22]^ preterm birth,^[20,21,23]^ macrosomia,^[22]^ and recently ICP.^[24–26]^ However, unlike the former study, a prospective study by Cui et al found no significant difference in the incidence of ICP between pregnant women with and without HBV infection.^[27]^

To investigate the possible association and estimated risk of ICP in patients with HBV infection, we conducted this meta-analysis to summarize all available evidence.

## 2 Materials and methods

### 2.1 Information retrieval

Four databases were searched from inception of the database to July 2019, including the Web of Science and EMBASE databases. The retrieval combined the following topic terms and free search terms: “hepatitis B or HBV” and “cholestasis in pregnancy or intrahepatic cholestasis of pregnancy or ICP. No language limitation was applied.

### 2.2 Inclusion and exclusion criteria

#### 2.2.1 Inclusion criteria

(1) Type of research: case-control, cross-sectional, or cohort studies.
(2) Research target: all original studies that evaluated the associations of ICP and HBV infection.
(3) Outcome index: odds ratios, relative risks, hazard ratios, or standardized incidence ratio with 95% confidence intervals (CI).

#### 2.2.2 Exclusion criteria

(1) Conference papers, reviews, lectures, abstracts, and other relevant published materials.
(2) Full-text papers unavailable.
(3) Study lacked significant information or with poor data integrity.
(4) Self-control clinical trials.

### 2.3 Document quality evaluation and data extraction

The quality of the studies was evaluated using the Cochrane risk of bias tool. Two researchers screened the studies, extracted the data, and evaluated the quality of the study based on the inclusion and exclusion criteria independently, using self-made data extraction forms, including basic information in the study, patient characteristics, and number of samples. Differences in the determination of study eligibility were resolved by consensus.

### 2.4 Statistical processing

Review Manager 5.3 (Cochrane Collaboration) was used to perform the meta-analysis. A fixed-effects or random-effects model was used for this meta-analysis. The statistical heterogeneity of the results of the studies was assessed using the Chi-squared test, expressed with the I^2^ index. If the heterogeneity test result was I^2^ ≤ 50%, it was deemed that there was no obvious heterogeneity in the included studies, and the fixed-effect model was used. When heterogeneity was detected, a possible explanation was pursued. If a reasonable cause was found, a subgroup analysis was then performed. Otherwise, a random-effects model was used. Variables are represented by the odds ratios (OR), and interval estimates are all based on the 95% confidence intervals. Differences were statistically significant at *P* < .05.

## 3 Results

### 3.1 Information retrieval results

The literature screening process and results are shown in Flow diagram. Initially, a total of 1008 related published articles were screened. Of those articles, 256 were first excluded due to duplicate publications. Seven hundred forty-three were excluded after reading the titles and abstracts and then 9 articles were further screened. Four of these cannot get 4-table and data of 1 article was not complete. After thorough reading and screening by layer, 4 studies were ultimately accepted for our investigation.

### 3.2 Basic characteristics and quality of selected literature

**Figure 1:**
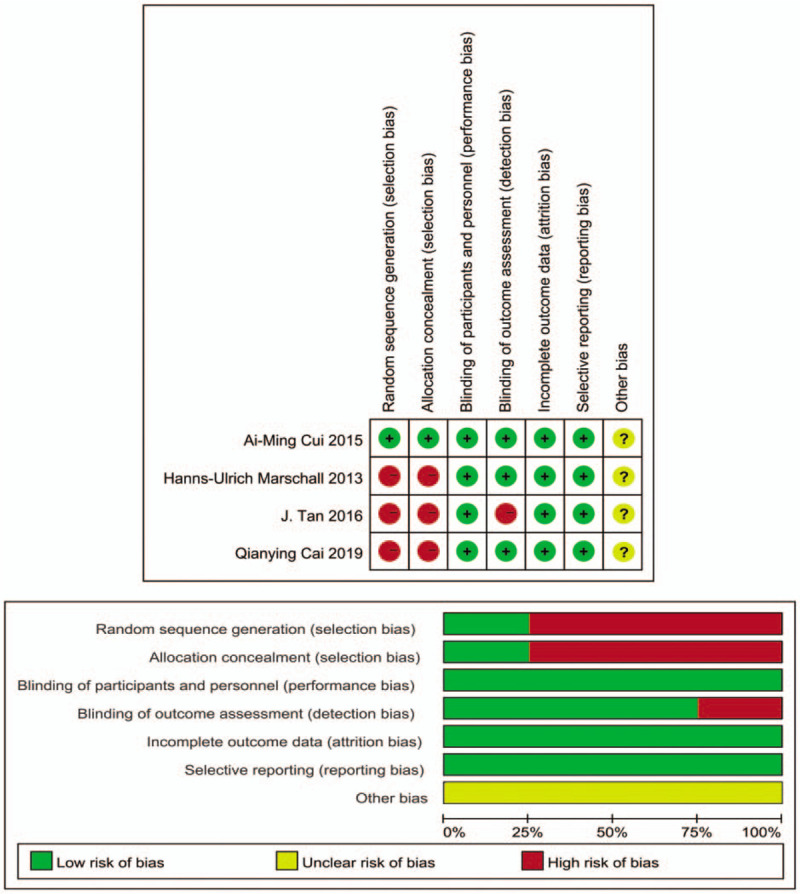
Risk of bias and clinical applicability of the included studies.

Table 1 summarizes the basic characteristics of the included studies. The 4 studies included 120,025 cases. Figure 1 show the results of the evaluation of literature quality. There were no selective reports or other sources of bias that were mentioned in the articles. Two articles were prospective cohort studies, one of these was hospital-based. One article was retrospective cohort study. Only 1 article was population-based cohort study.

**Table 1:**
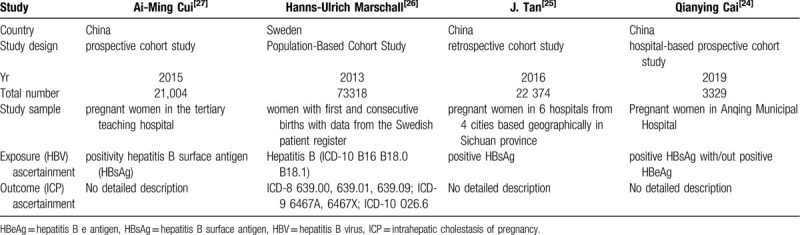
Basic characteristics of the included studies.

### 3.3 Systematic review results

**Figure 2:**
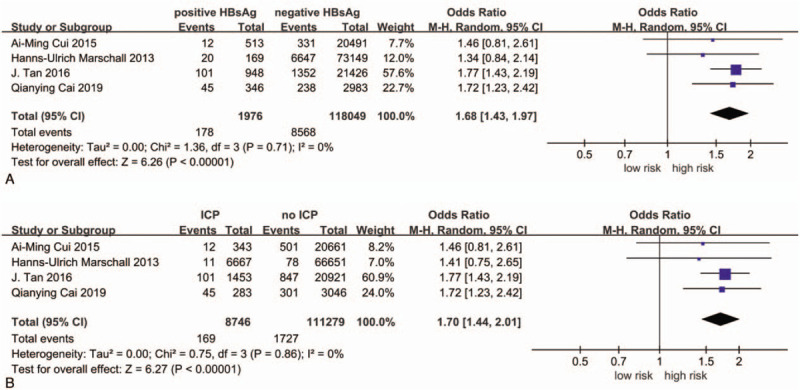
A: Forest plot of the included studies of the associations between hepatitis B infection and risk of intrahepatic cholestasis of pregnancy. B: Forest plot of the included studies of the associations between intrahepatic cholestasis of pregnancy and risk of hepatitis B infection.

Figure 2 Show the meta-analysis results.

For the first analysis, compared with non-HBV pregnant women, the OR of ICP in HBV-infected pregnant women was 1.68 (95% CI 1.43–1.97; I^2^ = 0%).

For the second analysis, compared with non-ICP patients, the OR of HBV infection among ICP patients was 1.70 (95% CI 1.44–2.01; I^2^ = 0%).

### 3.4 Publication bias evaluation

**Figure 3:**
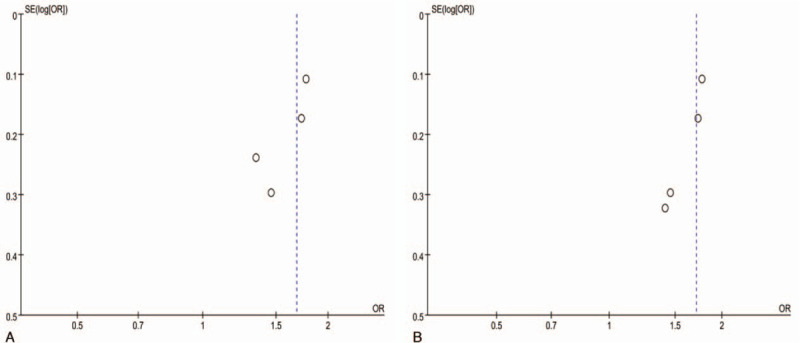
Funnel plot (A: between hepatitis B infection and risk of intrahepatic cholestasis of pregnancy; B: between intrahepatic cholestasis of pregnancy and risk of later hepatitis B infection).

There was no publication bias based on the funnel plot test results (Fig. 3).

## 4 Discussion

The aetiology of ICP is not fully understood, but it may be the result of a combination of genetic susceptibility (variants of hepatobiliary transport proteins), hormonal factors, and environmental factors.^[28–31]^ In addition, hepatitis virus infection may be a high-risk factor for ICP. Marschall et al analysed the data on 11,388 women with ICP and 113,893 women without ICP and found that women with ICP were more often diagnosed with hepatitis C (hazard ratio 4.16; 95% CI 3.14–5.51) compared with women without ICP (*P* < .001); later ICP was more common in women with pre-pregnancy hepatitis C (age-adjusted OR 5.76; 95% CI 1.30–25.44; *P* = .021).^[26]^ Similarly, Wijarnpreecha et al conducted a meta-analysis and found that the pooled OR of ICP in HCV-infected pregnant women compared with non-HCV pregnant women was 20.40 (95% CI 9.39–44.33); the pooled OR of later HCV infection among ICP patients compared with non-ICP patients was 4.08 (95% CI 3.13–5.31; I^2^ = 0%).^[32]^ They concluded that hepatitis C increased the risk of ICP. The American College of Obstetricians and Gynecologists, Royal College of Obstetricians and Gynaecologists, and the Society of Maternal Foetal Medicine note that patients who are seropositive for the hepatitis C virus may be at increased risk, though only the Society of Maternal Foetal Medicine gives an incidence (6%–16%).^[33–35]^ Recent studies have shown that HBV infection is associated with a higher incidence of ICP.^[24–26]^ Tan et al discovered that pregnant women positive for HBsAg had a higher risk of intrahepatic cholestasis (age-adjusted OR 1.74; 95% CI 1.40–2.16) and surmised that hepatitis B increased the risk of ICP.^[25]^ In a hospital-based prospective cohort study, Cai et al found that maternal HBsAg carrier status was associated with an increased risk of intrahepatic cholestasis pregnancy (age-adjusted OR 1.70; 95% CI 1.16–2.49); thus HBV infection during pregnancy may increase the risk of ICP.^[27]^

This study is the first systematic review and meta-analysis of published studies assessing the associations of ICP and HBV infection. We found that the risk of ICP was higher among pregnant women who were infected with HBV compared with those who were not.

Although the exact mechanism underlying this association between HBV infection and ICP is not understood, there are a few possible explanations. HBV is a hepatophilic virus that initiates infection by binding to receptors on the surface of hepatocytes. First, new data suggest that the liver injury in hepatitis B patients may affect natural killer cell function via PGE2.^[36]^ Some studies have described how HBV infection can modify the functions of several immune cells, such as natural killer cells, T cells, and granulocytic myeloid-derived suppressive cells, and lead to abnormal liver function and bile acid metabolism.^[37,38]^ Second, a recent study found a 12-fold increase in the incidence of ICP in sisters.^[39]^ Other studies have found that some genetic mutations are involved in the pathogenesis of ICP, such as the ABCB11,^[30,40]^ ABCC2,^[41]^ ABCB4,^[42,43]^ and NR1H4 coding sequences.^[31]^ These failures, along with high estrogen and progesterone levels during pregnancy, may increase the risk of ICP.^[44]^ Still other studies found that Na^+^-taurocholic acid co-transporting polypeptide (NTCP) is a functional receptor of hepatocytes infected with HBV, which mediates HBV invasion and infection by binding specifically with HBV envelope protein pre-S1 antigen, and is linked with the mechanism of HBV infection.^[45–47]^ Na^+^-taurocholic acid co-transporting polypeptide is the main hepatic transporter of conjugated bile acids, and the entry receptor for HBV, which along with the altered hormone levels during pregnancy may increase the risk of ICP among women infected with HBV.

Unlike hepatitis C virus infection, the studies included in our analysis mainly examined carriers of HBV or patients with chronic hepatitis B, and few people were infected with HBV during pregnancy. Interestingly, we also found that compared with pregnant women without ICP, the incidence of HBV infection in pregnant women with ICP was significantly higher. This suggests that clinicians should routinely perform hepatitis virus tests, including HBV and other hepatitis viruses, in patients with ICP, especially in pregnant women who have itchy skin and are diagnosed with ICP early-mid trimester.

Researchers are also concerned with the correlation between the activity of HBV and ICP. Furthermore, Cai et al focused on hepatitis B e antigen (HBeAg) and found that the risk of ICP is higher in HBeAg carriers than in HBeAg-negative pregnant women.^[27]^ Some studies have noted that among the different phases of HBV infection, the immune-clearance phase positive for HBeAg showed less severe histological activity and harboured fewer mutations than that when negative for HBeAg, implying that HBeAg increases the risk of ICP by increasing the impact of HBV on bile acid metabolism via the immune response.^[48–51]^ However, the research on HBeAg and ICP is limited, and more studies are needed.

HBV covalently closed circular DNA, a plasmid-like episome in the host cell nucleus from the protein-linked relaxed circular DNA genome in incoming virions, is a marker of viral replication that is associated with abnormal liver function.^[52–54]^ Approximately 15 million HBV patients have developed hepatitis delta virus infections in addition to their HBV infections.^[55]^ The patients superinfected with this satellite virus develop more severe disease.^[56,57]^ However, there are few articles on the risk of ICP in patients with HBV covalently closed circular DNA or hepatitis delta virus, and more research on this is needed.

Although most of the included studies were of high quality as reflected by the evaluation scores, this meta-analysis has some limitations, and the results should be interpreted with caution. First, the number of studies included was too small to assess publication bias; therefore, publication bias in favour of positive research may already exist. Second, all other biological features that may affect the outcome might not be included. Third, most of the included studies used databases based on medical registries, increasing the likelihood of inaccurate coding. In addition, this is a meta-analysis of observational studies, which can only prove an association, and cannot establish a causal relationship.

In summary, this meta-analysis found an increased risk of ICP among HBV-infected pregnant women, but also an increased risk of HBV infection among ICP patients. This suggests that to avoid adverse pregnancy outcomes, clinicians should strengthen the screening for ICP in pregnant women with HBV and improve the monitoring of serum bile acid and liver function in patients. In addition, pregnant women diagnosed with ICP should be tested routinely for HBV infection.

## Author contributions

JR is the guarantor. All review authors critically reviewed, revised, and approved the subsequent and final version of the protocol.

**Conceptualization:** Jiang Ruoan,

**Data curation:** Wang Ting, Zhou Feifei.

**Formal analysis:** Jiang Ruoan, Yao Yingsha.

**Methodology:** Jiang Ruoan, Wang Ting.

**Project administration:** Huang Xiufeng.

**Software:** Yao Yingsha.

**Supervision:** Huang Xiufeng.

**Validation:** Wang Ting.

**Writing:** Jiang Ruoan, Yao Yingsha.
